# Computed Tomography Angiography as Ancillary Testing for Death Determination by Neurologic Criteria: A Technical Review

**DOI:** 10.3390/tomography10070086

**Published:** 2024-07-16

**Authors:** Abanoub Aziz Rizk, Jai Shankar

**Affiliations:** 1 Faculty of Medicine, University of Ottawa, Ottawa, ON K1H 8M5, Canada; arizk082@uottawa.ca; 2 Department of Radiology, University of Manitoba, Winnipeg, MB R3C 2C1, Canada

**Keywords:** CT angiogram, brain death, death by neurological criteria

## Abstract

The determination of death by neurological criteria (DNC) stands as a pivotal aspect of medical practice, involving a nuanced clinical diagnosis. Typically, it comes into play following a devastating brain injury, signalling the irreversible cessation of brain function, marked by the absence of consciousness, brainstem reflexes, and the ability to breathe autonomously. Accurate DNC diagnosis is paramount for adhering to the ‘Dead donor rule’, which permits organ donation solely from deceased individuals. However, complexities inherent in conducting a comprehensive DNC examination may impede reaching a definitive diagnosis. To address this challenge, ancillary testing such as computed tomography angiography (CTA) has emerged as a valuable tool. The aim of our study is to review the technique and interpretation of CTA for DNC diagnoses. CTA, a readily available imaging technique, enables visualization of the cerebral vasculature, offering insights into blood flow to the brain. While various criteria and scoring systems have been proposed, a universally accepted standard for demonstrating full brain circulatory arrest remains elusive. Nonetheless, leveraging CTA as an ancillary test in DNC assessments holds promise, facilitating organ donation and curbing healthcare costs. It is crucial to emphasize that DNC diagnosis should be exclusively entrusted to trained physicians with specialized DNC evaluation training, underscoring the importance of expertise in this intricate medical domain.

## 1. Death Determination by Neurological Criteria

The determination of death by neurological criteria (DNC) is defined as permanent cessation of brain function characterized by the absence of consciousness, brainstem reflexes, and the ability to breathe independently [1,2,3]. The occurrence of DNC is always linked to a devastating brain injury, but various sources have different approaches that accurately explain its complexities. DNC is, however, always defined as a lack of responsiveness, respiratory movements, and brain stem activity [4,5]. DNC is a clinical diagnosis. Confounding factors of DNC, such as unresuscitated shock, hypothermia, facial trauma, high cervical spinal cord injury, and other factors, can affect the clinician’s ability to perform a complete clinical DNC exam and the validity of the clinical results without ambiguity [6,7,8]. Even if the criteria for DNC are met, some patients may continue to manifest spontaneous reflexive movements, which can cause ambiguity and apprehension for clinicians and families in accepting the diagnosis of DNC [1,5]. Furthermore, some patients could have false positive triggering of the ventilator, which could also impact the diagnosis of DNC [1,6]. Following the determination of DNC, the patient is deemed deceased, and artificial life support may be terminated. Many of these patients potentially could be organ donation candidates. A definite determination of death is required to comply with the ‘Dead donor rule’, which mandates that organ donation can only occur from a deceased patient [9,10]. In several jurisdictions, this requires an ancillary test to confirm a diagnosis of death [1,2,11]. Therefore, a very rigorous assessment of the physician’s suspicion regarding DNC must be made to eliminate errors with such an important diagnosis. 

## 2. Clinical Diagnosis of DNC

The clinical diagnosis of DNC is always made by a trained physician, such as an intensivist, who has received training in DNC evaluation [12]. DNC is often described as requiring three major conditions: (1) deep unresponsiveness or a Glasgow Coma Scale (GCS) less than 3; (2) the inability to breathe independently; and (3) absent cranial nerve reflexes including pupillary light, corneal, oculo-cephalic, cough, and/or gag reflexes [12].

A painful stimulus is also always required, systematically applied centrally and peripherally, above and below the neck. A verbal response is never possible during a DNC examination because the patient is invariably intubated [1,5,13].

The next step is for the clinicians to conduct an apnea test, which entails temporarily removing any mechanical ventilation support and observing for any spontaneous breathing [6]. The apnea test is a medical procedure that only needs to be performed once if the results are conclusive. However, prior to conducting the test, the physician must ensure that the patient meets certain conditions, which include a core temperature of at least 36.5 °C, or 97.7 °F, euvolemia (with the option of a positive fluid balance in the previous 6 h), a normal PCO2 (with the option of an arterial PCO2 of at least 40 mm Hg), and a normal PO2 (with the option of pre-oxygenation to an arterial PO2 of at least 200 mm Hg). These conditions must be met to ensure accurate results from the apnea test [6].

Overall, to conduct a proper clinical evaluation of DNC, it is crucial to verify the presence of a clear cause, such as radiographic proof of severe brain injury accompanied by indications of trans-tentorial herniation while also ruling out any potential confounding factors.

## 3. Ancillary Testing

While the implementation of ancillary testing has been deemed mandatory in some countries around the world, other countries continue to not require an additional workup [1]. Overall, the use of instrumental testing to confirm a diagnosis of DNC continues to be a subject of debate. An international survey showed that 41% of European countries required mandatory ancillary testing for the diagnosis of DNC [1]. The reasons for such global protocol discrepancies could be due to different apnea testing protocols, a time lag due to long wait times prior to the assessment of DNC, simply physician preference, a trend towards increasing the use of imaging tests, and different guidelines to diagnose DNC [1]. One key advantage of confirmatory testing is the ability to establish an early, objective, and accurate diagnosis, which can decrease the risk of an incorrect call. This can be especially important in critically ill patients to optimise their management. In cases where a patient is declared brain-dead and a candidate for organ donation, timely and accurate diagnosis is crucial to ensure that organs are properly preserved and can be transplanted to waiting recipients. This not only saves lives but also helps to ease the burden on our limited healthcare resources, including the need for unnecessary or redundant procedures. For these ancillary imaging tests to be used for the determination of DNC, their accuracy and reliability must be quite high and well understood. While studies have explored the accuracy of different ancillary testing modalities, there is no consensus on which modality has the highest accuracy. Nevertheless, ancillary tests are indicated when a clinical determination cannot be made because of confounding factors or physiologic instability.

Diagnosing DNC requires comprehensive and conclusive evidence of the irreversible cessation of all brain activity. Several ancillary imaging techniques have been described for the diagnosis of DNC, including electroencephalogram (EEG), conventional catheter angiograms [14], computer tomography angiogram (CTA), magnetic resonance angiogram, nuclear blood flow scan, and transcranial Doppler (TCD) [3]. Scintigraphy, especially with the use of technetium-99m hexamethylpropyleneamine oxime (HMPAO), is a nuclear medicine imaging technique that assesses cerebral blood flow. During the procedure, the radiotracer is injected into the bloodstream, and images are captured to show areas of perfusion. In the context of DNC, the absence of tracer uptake in the brain—termed the “hollow skull” or “empty light bulb” sign—indicates a lack of cerebral blood flow, thereby supporting the diagnosis. Magnetic resonance imaging (MRI) offers a detailed and high-resolution view of brain structures and is particularly useful in identifying conditions such as brain edema, herniation, and global cerebral ischemia, all of which are consistent with DNC. Diffusion-weighted imaging (DWI) and magnetic resonance angiography (MRA) are specific MRI techniques that can detect cytotoxic edema and confirm the absence of intracranial blood flow, respectively. The comprehensive imaging capabilities of MRI allow for a thorough assessment of brain tissue integrity and perfusion, making it a critical tool in confirming DNC when clinical tests alone are inconclusive or when additional confirmation is required. Doppler ultrasound, specifically transcranial Doppler (TCD), is a non-invasive technique that measures cerebral blood flow velocity in the major brain arteries. In a diagnosis of DNC, TCD can reveal characteristic changes in blood flow patterns, such as the absence of diastolic flow or the presence of reverberating flow and small systolic spikes, which are indicative of severely reduced or absent cerebral perfusion. The simplicity and bedside applicability of Doppler ultrasound makes it a valuable tool for continuous monitoring and repeated assessments in critically ill patients. Together, scintigraphy, MRI, and Doppler ultrasound provide a robust, multi-modal approach to diagnosing DNC, each contributing unique and complementary insights that ensure a reliable and accurate determination of this critical condition.

CT-based diagnostic imaging techniques are becoming widely used due to their availability, relatively lower cost, speed, non-invasive methods, and technical experience in performing these tests. Of these, CTA has become one of the most widely used ancillary imaging tests for this indication. The purpose of this review is to appraise the full scope of using CTA as an ancillary imaging test to assess DNC, including the indication, technique, findings, advantages, and disadvantages of CTA as an ancillary imaging test for DNC determination.

## 4. CTA in Brain Death Diagnosis

CTA is an imaging modality commonly used worldwide, serving as the primary vascular imaging test to assess active flow in the blood vessels [1,15]. Although CTA provides 2D static images, opacification of blood vessels by the CT contrast agent is considered equivalent to the presence of active flow. While CTA offers a comprehensive assessment of vessels of various sizes, it does not provide information on capillary-level flow. Opacification of arteries and veins in a specific circulation system serves as a surrogate for the patency of the intervening capillary network they support. This holds true in simpler circulation systems with one or two arterial supplies and venous drainage but becomes challenging in the brain, which features multiple collateral arterial supplies and several venous drainage pathways.

**Figure 1:**
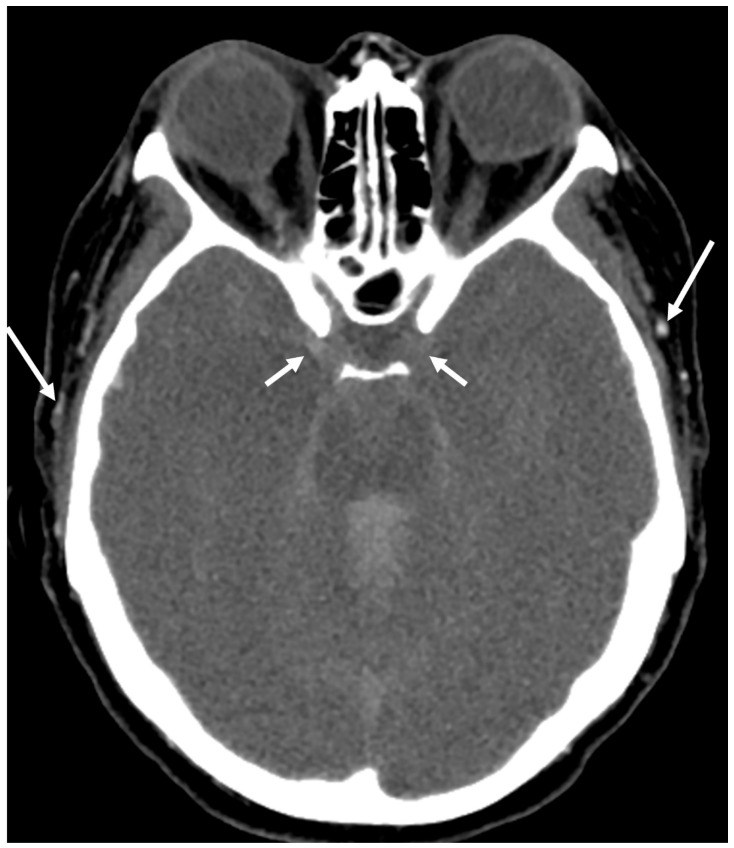
CTA showing classic imaging findings for confirmation of DNC with no opacification of intracranial internal carotid arteries (short arrows) and continued opacification of external carotid branches (long arrows).

CTA has also garnered interest as a method for diagnosing DNC, primarily due to its feasibility, wide availability, non-invasiveness, relatively low cost, and expeditiousness [1,15]. Studies from across the world have reported the efficacy of using CTA for DNC diagnosis. Various imaging protocols and assessment criteria have been described for DNC determination. These protocols are based on the principle of non-opacification of intracranial arteries and veins by the CT contrast in cases of DNC (Figure 1). The utilization of CTA as a supplementary imaging modality rests on the fundamental premise that cerebral blood flow is critical for brain tissue viability, with the presence of blood flow within major intracranial vessels serving as a surrogate marker for viable neurons. However, the absence of blood flow could persist at a later stage due to a lack of metabolic demand, although this area of inquiry remains inadequately explored. A diagnosis of DNC using CTA assumes the uniform global death of brain cells in patients with DNC, and DNC can only be diagnosed when there is a global absence of blood flow in the brain blood vessels. Absence of blood flow only in a small part of the brain, as in cases of ischemic stroke, should not be considered DNC. Any presence of intracranial blood flow, especially in the distal arterial system and venous system, precludes the diagnosis of DNC.

However, the main deterrent to the use of CTA in DNC determination is the lack of diagnostic confidence due to the absence of standardized technical protocols and interpretation criteria.

## 5. CTA Imaging Protocol

CTA images are acquired during the intravenous injection of CT contrast agent, typically ranging from 65 to 120 mL at a rate of 3 to 5 milliliters per second. The thickness utilized in these CTA acquisitions ranges from 0.5 to 0.9 mm, with reconstruction increments of 0.33 to 0.45 mm [6]. The iodinated contrast is administered intravenously, preferably through an 18G IV canula, into the antecubital vein using a power injector at 4 mL per second, followed by a saline flush at the same rate [6]. On previous-generation scanners, CTA consistently displayed opacification of the intracranial venous system. However, on the current-generation CT scanners, intracranial venous system opacification may not be evident due to the much faster speed of image acquisition. To address the potential lack of venous opacification on CTA, a two-phase CTA is recommended for DNC diagnosis. In this approach, contrast is injected only once during the acquisition of the first-phase standard CTA images. Second-phase CTA images are acquired 60 s after the start of the first-phase CTA acquisition without additional contrast injection. This delayed acquisition ensures contrast opacification of the intracranial venous system. While the delayed venous phase is advantageous for confirming proper venous system opacification, it also enables passive diffusion of contrast into the intracranial arterial system over time. This can pose challenges, as it may lead to false opacification of the arterial system in the absence of intracranial flow. Therefore, while the delayed phase aids in confirming venous system opacification, it also increases the potential for false opacification of intracranial arteries.

## 6. CTA Scoring Systems

**Figure 2:**
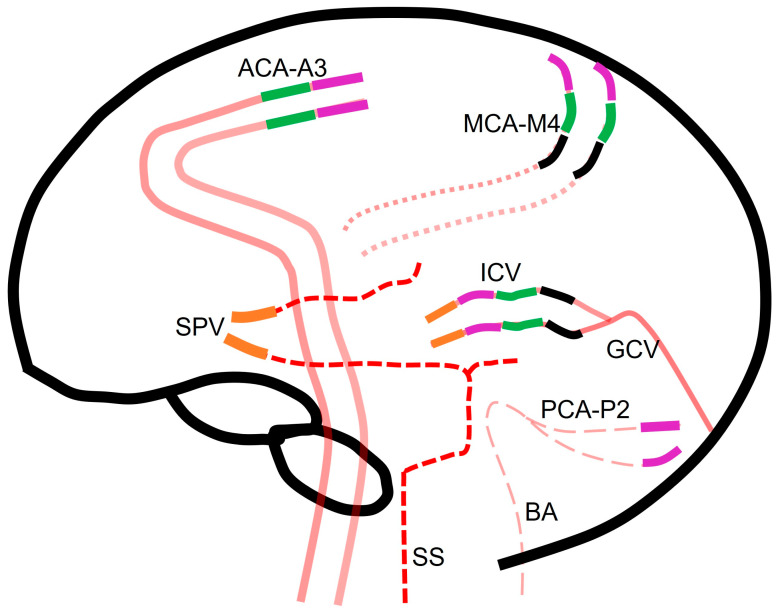
Black: 4-point system assigns points for non-opacifications of distal vessels such as M4 branches of the middle cerebral arteries and the internal cerebral veins. Orange: venous score assigns points for non-opacification of both the internal cerebral veins and the superior petrosal veins. Purple: 10-point system assigns points based on non-opacification of the bilateral middle cerebral artery (M4), the bilateral internal cerebral veins, the bilateral posterior cerebral arteries (P2), the basilar artery, the bilateral anterior cerebral artery (A3), and the great cerebral vein. Green: 7-point system assigns points for non-opacification of the distal MCA branches, pericallosal arteries, internal cerebral veins, and great cerebral veins. Red dashed line represents the venous system of transverse and sigmoid sinuses; pink solid line represents internal carotid artery; pink dotted line represent middle cerebral arteries and pink dashed line represent the vertebral, basilar and posterior cerebral arteries.

Several scoring systems have been proposed for interpretating CTA findings for DNC diagnosis, with no consensus on superiority. These scores are typically assessed during the second phase of a two-phase CTA, as single-phase CTAs are deemed insufficient for DNC diagnosis [5]. Table 1 and Figure 2 summarize different scoring systems used for diagnosing DNC via CTA and the different blood vessel opacifications assessed in each system.

**Figure 3:**
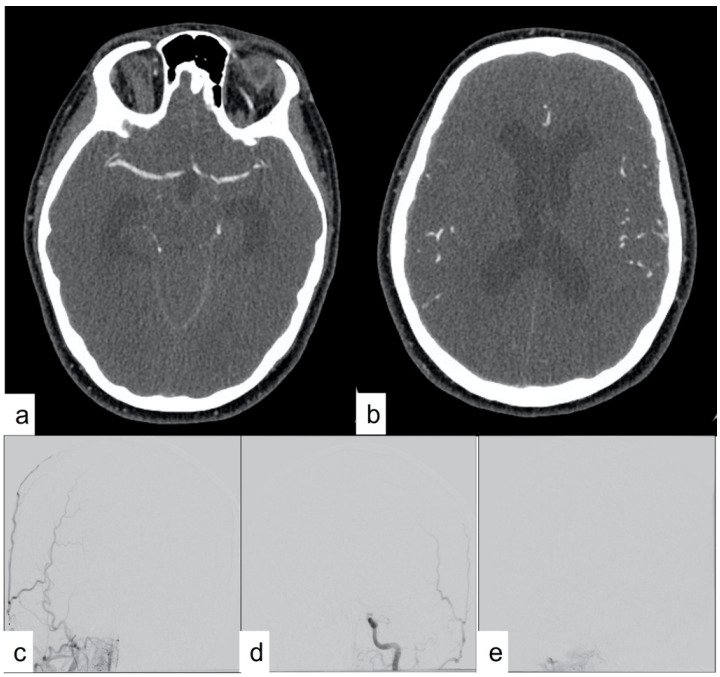
CTA showing opacification of intracranial arteries but no opacification of the deep venous system (**a**,**b**). On the same day, a digital subtraction angiogram of the right (**c**) and left (**d**) common carotid arteries and dominant right vertebral artery (**e**) showed no filling of intracranial arteries but continued filling of the extracranial arteries, consistent with findings of DNC. This is based on most of the scoring system criteria, but DNC cannot be confirmed except using the venous score.

All criteria require confirmation of opacification of external carotid artery branches to verify intravascular contrast injection. Points are assigned in the four CTA scoring systems as follows:

- In the 4-point system, one point is assigned for each instance of non-opacifications of the M4 branches of the middle cerebral arteries (MCA) and the internal cerebral veins (ICV) on each side.
- The 7-point system assigns one point each for non-opacification of the M4 branches of the MCAs, pericallosal arteries, and ICVs on each side and great cerebral vein.
- Within the 10-point system, one point is allotted for non-opacification of the M4 branches of the MCAs, ICVs, the P2 segment of bilateral posterior cerebral arteries, the basilar artery, the A3 segment of bilateral anterior cerebral artery (pericallosal artery), and the great cerebral vein.
- The venous score assigns one point each for non-opacification of both the ICVs and the superior petrosal veins (Figure 3).

While most scoring systems prioritize the evaluation of both arterial and venous systems, venous scoring systems focus exclusively on assessing the filling of the deep venous system. The principle underlying this approach is that if the brain maintains sufficient blood flow for viability, contrast flow from the arterial to the venous side should be detectable. Although the superficial venous system could potentially fill from the extracranial circulation, the deep venous system relies solely on intracranial circulation for adequate filling. Any opacification of the venous system precludes a diagnosis of DNC (Figure 3). Hence, an adequate venous phase is critical for the diagnosis of brain death. This raises questions about the necessity of arterial opacification in any of the scoring systems. While no scoring system has proven superior to others, it is intuitive that the more vessels included in the scoring system, the lower its sensitivity will be in diagnosing DNC. This is crucial because the purpose of an ancillary test is not only to diagnose DNC but also to rule out its absence. A scoring system that includes a larger number of blood vessels is more reliable regarding falsely declaring someone brain dead, whereas a scoring system that includes fewer blood vessels is simpler and likely more accurate in confirming DNC.

Studies have shown that the sensitivity of CTA was 62% when there was an absence of any opacifications of blood vessels supplying the brain during the venous phase, and the sensitivity increased to 84% for CTAs performed during the arterial phase [1]. However, a concern arises, as many patients diagnosed with DNC clinically or radiographically continue to have some opacifications of the proximal intracranial arteries (but not of the veins) on CTA [1]. Due to this incomplete intracranial circulatory arrest, studies have advised against using CTA to diagnose DNC when an additional clinical declaration using a test such as the apnea test cannot be completed [1]. However, this again raises the need for arterial opacification in any of the scoring systems, perpetuating the debate.

## 7. The Challenge in Pediatrics

Diagnosing DNC within the pediatric population is typically performed by pediatric intensive care physicians and neurologists [16]. Multiple ancillary testing modalities are used in the pediatric populations, similar to those in adults. While CTA imaging is widely available, it is often met with uncertainty when used to diagnose DNC in pediatric patients. A study showed that 94% of CTAs utilized as ancillary testing for the diagnosis of DNC confirmed the diagnosis. Additionally, in 86% of patients, the EEG, TCD, and CTA findings were all consistent with the clinical determination [16]. The use of CTA in the pediatric population presents notable challenges. Despite its widespread availability, CTA is infrequently utilized in younger children, resulting in a limited experience with the modality in this age group. Additionally, the relatively high baseline heart rate observed in pediatric patients compared to adults can pose difficulties in accurately capturing the contrast bolus post injection with existing software, leading to venous contamination. As previously mentioned, any form of venous contrast opacification can obscure the diagnosis of DNC. However, it is essential to note that due to factors such as lack of experience, technical complexities, and concerns regarding radiation and contrast exposure, CTA is not the preferred modality for vascular imaging in the pediatric population. Nonetheless, with increased experience in utilizing CTA in pediatric patients, there is hope that these challenges can be effectively addressed in the future.

## 8. Limitations of CTA as an Ancillary Test

Despite several benefits, CTA as an ancillary test also presents limitations [12]. One significant issue in diagnosing DNC with CTA involves the challenge of assessing intracranial filling of small distal intracranial arteries and veins [15]. Some patients, particularly those with subarachnoid hemorrhages or an increased density of the subarachnoid space due to hemorrhages, may present difficulties in determining the level of intracranial opacification, leading to discrepancies between radiologists who are interpreting the CTA images [15].

The concept of isolated brainstem death contradicts the initial notion that DNC is a global phenomenon. In a small subset of patients clinically confirmed with DNC, there may be continued opacification of intracranial blood vessels [17]. These patients exhibit isolated brainstem death with absent perfusion in the brainstem but continued perfusion in the rest of the brain parenchyma, making them ineligible for a DNC diagnosis solely using CTA. A computer tomography perfusion (CTP) study showed utility in correctly diagnosing isolated brainstem death in such cases [5,18].

When evaluating CTA for DNC assessment, confirming intravascular injection of contrast is imperative to avoid a false DNC determination. Malfunctions of injector pumps, interstitial contrast injections, or accidental saline injections can lead to erroneous conclusions. Verification of contrast opacification of external carotid artery (ECA) branches on CTA serves as a crucial quality check for all CTAs, especially when employed as an ancillary test for DNC confirmation.

## 9. Apprehension of Radiologists

The increased use of CTA to confirm DNC has enhanced radiologists’ comfort regarding the interpretation of these images. However, this heightened responsibility also amplifies apprehension among radiologists, particularly when clinical information is limited. Faced with the challenging task of declaring DNC in a patient, radiologists often experience significant apprehension and concern due to their weighty responsibility. It is crucial for radiologists to recognize that the final diagnosis of DNC relies on clinical criteria. Ancillary tests, including CTA, are employed only when clinical criteria cannot be fully assessed. Therefore, CTA findings constitute just one piece of the broader clinical puzzle in DNC diagnosis. They should never be used in isolation for DNC diagnosis; instead, they serve as confirmatory tests. Additionally, in complex scenarios where radiologists struggle to provide a conclusive diagnosis, it is prudent to err on the side of caution to prevent errors. In such cases, collaboration with the clinical team is essential to determine the most appropriate use of ancillary test findings, optimizing the utility of CTA as a valuable adjunctive tool for DNC confirmation.

## 10. Conclusions

The determination of DNC is a clinical diagnosis but can be challenging. To address this challenge, ancillary testing using CTA has emerged as a valuable tool, as it is a readily available imaging technique and enables visualization of the cerebral vasculature, offering insights into blood flow to the brain. Leveraging CTA as an ancillary test in DNC assessments holds promise because it can facilitate organ donation and curb healthcare costs.

## Figures and Tables

**Table 1:** Summary of CTA scoring systems for death determination by neurological criteria.

	M4 (R and L)	A3 (R and L)	BA	P2 (R and L)	ICV (R and L)	VOG	SPV	Total Score
4 point	2	*	*	*	2	*	*	4
7 point	2	2	*	*	2	1	*	7
10 point	2	2	1	2	2	1	*	10
VS	*	*	*	*	2	*	2	4

Legend: M4 (segment 4 of the middle cerebral artery); A3 (segment 3 of the anterior cerebral artery); BA (basilar artery); P2 (segment 2 of posterior communicating artery); ICV (internal cerebral vein); VOG (great cerebral vein of Galen); VS (venous score); SPV (superior petrosal vein); L (left); and R (right); * not applicable for the scoring system.
